# Avoiding Sutured Tie-Over Dressings on the Nasal Dorsum

**DOI:** 10.1155/2010/958213

**Published:** 2010-06-21

**Authors:** A. Harb, A. N. Pandya

**Affiliations:** Department of Plastic Surgery, Queen Alexandra Hospital, Portsmouth, Hampshire PO6 3LY, UK

## Abstract

The nose is a common site for basal and squamous cell carcinomas. Surgical excision can leave a defect that is appropriate for reconstruction with full thickness skin grafting. Tie-over dressings have gained popularity in this setting. We propose that steri-strips alone provide the necessary support to facilitate excellent graft take.

## 1. Introduction

Nonmelanoma skin cancer is the most common cancer in the United Kingdom. It is estimated that in 1999 there were more than 59,000 new cases diagnosed [1]. Basal cell carcinoma (BCC) constitutes seventy five percent of all nonmelanoma skin cancers. BCCs occur at different anatomical sites with eighty percent occurring on the head and neck [2]. There is preponderance for sun exposed areas and particularly for more prominent features such as the nose. 

Surgical excision has an important role in the management of patients with BCCs. Adequate excision of these lesions often leaves a defect for which full thickness skin grafting is an appropriate reconstructive modality. Seymour (2003) cites Blair and Brown (1929) [3] as stressing that one of the basic requirements for successful grafting was the application of even pressure to a skin graft by a carefully designed dressing. Tie-over dressings have gained popularity over the years in the belief that they are the best way of achieving this. There have been many varieties of tie-over conceived [4–6]. In the recent study of the pressure created by tie-over dressings, Seymour and Giele (2003) [3] showed that no significant pressure was created by the tie-over dressing. Previous work by Davenport (1988) [7] also concluded that the concept of “pressure” from a bolus tie-over dressing being necessary for the successful “take” of a full thickness skin graft is erroneous. We believe that it is probably immobility that optimises skin graft survival. On the nasal dorsum, steri-strips applied correctly can mould a dressing to the contour of the region sufficiently to allow good graft contact across its bed and facilitate excellent take. 

## 2. Technique

**Figure 1:**
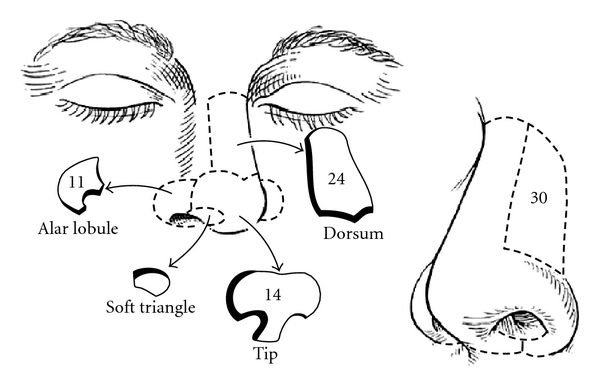
A diagram showing the regions of the nose and the incidence of non-melanoma skin cancers in our study. Lateral nasal aspect (30), nasal dorsum (24), nasal tip (14), and alar region (11).

A series of 79 patients requiring grafting of a defect on the bridge of their nose by the senior author were added to the study between January 2001 and July 2008. The majority of the lesions were over the lateral nasal aspect (30), followed by the nasal dorsum (24), the nasal tip (14), and the alar region (11) (Figure 1). 

Standard excisions were performed under the appropriate anaesthetic. All defects were skin grafted immediately after excision of the primary malignancies. Full thickness grafts were harvested from the supraclavicular or postauricular areas. The grafts were sutured to their bed circumferentially with 5/0 monofilament nylon for the first seven patients and with 5/0 or 6/0 vicryl rapide for the remainder. 6/79 patients had quilting sutures using 4/0 or 5/0 vicryl rapide. A single sheet of Jelonet was applied over the graft and held in place with steri-strips. None of the patients had any additional foam dressing. All dressings were examined between the fifth and seventh post operative days, usually at the time of first followup.

## 3. Results

A total of 79 non-melanoma skin cancer excisions were performed, (56 BCC; 23 SCC), followed by full thickness skin grafting. The average size of the defect was 31 mm (20–53 mm). Three of the 79 patients had a slight reddening at the graft periphery. Two of these patients had previously had ulcerated lesions and they were treated with antibiotics. One patient showed a suspected reaction to the dissolvable suture material, which was subsequently removed. There has been no incidence of graft loss in any of these patients.

## 4. Discussion

The dressing applied to a full thickness graft is designed to provide an environment to facilitate 100% graft take. It must protect against accidental trauma and prevent shear of the graft on its bed. It must apply sufficient pressure to keep good graft-to-bed contact. It should minimise bacterial colonisation. It is advantageous if the dressing is not too bulky or limiting to the patient. Our series shows that the simple application of steristrips will achieve all of these objectives.

The hypothesised mechanism of action of the tie-over pressure dressing has shown to be flawed. Seymour (2003) showed that the tie-over dressing did not create the 25 mmHg of pressure required to prevent seroma and haematoma formation [3]. It is hypothesised in their paper that it is the prevention of shear that is the mechanism of action of the tie-over dressing. We have shown that our technique is able to stabilise the graft/bed interface as effectively as possible.

The commonly used sutured tie-over dressing can be a relatively lengthy dressing to construct. The use of a single sheet of Jelonet secured with Steristrips, and avoidance of additional foam and sutures, make this dressing much less bulky to apply and more rapid to construct, with no detrimental effects on graft survival.

The vast array of tie-over dressing techniques will continue to have their place in surgical practice. Our experience highlights that in the right situation the more complicated and fiddly methods can be swapped for a simple steristrip technique, which is quick and effective, with no adverse effects.
